# Ypel5 regulates liver development and function in zebrafish

**DOI:** 10.1093/jmcb/mjad019

**Published:** 2023-03-22

**Authors:** Yun Deng, Xiao Han, Huiqiao Chen, Chaoxian Zhao, Yi Chen, Jun Zhou, Hugues de The, Jun Zhu, Hao Yuan

**Affiliations:** Shanghai Institute of Hematology, State Key Laboratory of Medical Genomics, National Research Center for Translational Medicine at Shanghai, Ruijin Hospital, Shanghai Jiao Tong University School of Medicine, Shanghai 200025, China; CNRS-LIA Hematology and Cancer, Sino-French Research Center for Life Sciences and Genomics, Ruijin Hospital, Shanghai Jiao Tong University School of Medicine, Shanghai 200025, China; Shanghai Institute of Hematology, State Key Laboratory of Medical Genomics, National Research Center for Translational Medicine at Shanghai, Ruijin Hospital, Shanghai Jiao Tong University School of Medicine, Shanghai 200025, China; CNRS-LIA Hematology and Cancer, Sino-French Research Center for Life Sciences and Genomics, Ruijin Hospital, Shanghai Jiao Tong University School of Medicine, Shanghai 200025, China; Department of Hematology, Sir Run Run Shaw Hospital, Zhejiang University School of Medicine, Hangzhou 310011, China; Shanghai Institute of Hematology, State Key Laboratory of Medical Genomics, National Research Center for Translational Medicine at Shanghai, Ruijin Hospital, Shanghai Jiao Tong University School of Medicine, Shanghai 200025, China; CNRS-LIA Hematology and Cancer, Sino-French Research Center for Life Sciences and Genomics, Ruijin Hospital, Shanghai Jiao Tong University School of Medicine, Shanghai 200025, China; Shanghai Institute of Hematology, State Key Laboratory of Medical Genomics, National Research Center for Translational Medicine at Shanghai, Ruijin Hospital, Shanghai Jiao Tong University School of Medicine, Shanghai 200025, China; Shanghai Institute of Hematology, State Key Laboratory of Medical Genomics, National Research Center for Translational Medicine at Shanghai, Ruijin Hospital, Shanghai Jiao Tong University School of Medicine, Shanghai 200025, China; CNRS-LIA Hematology and Cancer, Sino-French Research Center for Life Sciences and Genomics, Ruijin Hospital, Shanghai Jiao Tong University School of Medicine, Shanghai 200025, China; CNRS-LIA Hematology and Cancer, Sino-French Research Center for Life Sciences and Genomics, Ruijin Hospital, Shanghai Jiao Tong University School of Medicine, Shanghai 200025, China; Université de Paris 7/INSERM/CNRS UMR 944/7212, Equipe Labellisée Ligue Nationale Contre le Cancer, Hôpital St. Louis, Paris 75010, France; CNRS-LIA Hematology and Cancer, Sino-French Research Center for Life Sciences and Genomics, Ruijin Hospital, Shanghai Jiao Tong University School of Medicine, Shanghai 200025, China; Université de Paris 7/INSERM/CNRS UMR 944/7212, Equipe Labellisée Ligue Nationale Contre le Cancer, Hôpital St. Louis, Paris 75010, France; Shanghai Institute of Hematology, State Key Laboratory of Medical Genomics, National Research Center for Translational Medicine at Shanghai, Ruijin Hospital, Shanghai Jiao Tong University School of Medicine, Shanghai 200025, China; CNRS-LIA Hematology and Cancer, Sino-French Research Center for Life Sciences and Genomics, Ruijin Hospital, Shanghai Jiao Tong University School of Medicine, Shanghai 200025, China

**Keywords:** YPEL5, hepatomegaly, hepatic function, HNF4A, PPARα signaling

## Abstract

*YPEL5* is a member of the Yippee-like (YPEL) gene family that is evolutionarily conserved in eukaryotic species. To date, the physiological function of YPEL5 has not been assessed due to a paucity of genetic animal models. Here, using CRISPR/Cas9-mediated genome editing, we generated a stable *ypel5*^−/−^ mutant zebrafish line. Disruption of *ypel5* expression leads to liver enlargement associated with hepatic cell proliferation. Meanwhile, hepatic metabolism and function are dysregulated in *ypel5*^−/−^ mutant zebrafish, as revealed by metabolomic and transcriptomic analyses. Mechanistically, Hnf4a is identified as a crucial downstream mediator that is positively regulated by Ypel5. Zebrafish *hnf4a* overexpression could largely rescue *ypel5* deficiency-induced hepatic defects. Furthermore, PPARα signaling mediates the regulation of *Hnf4a* by Ypel5 through directly binding to the transcriptional enhancer of the *Hnf4a* gene. Herein, this work demonstrates an essential role of Ypel5 in hepatocyte proliferation and function and provides the first *in vivo* evidence for a physiological role of the *ypel5* gene in vertebrates.

## Introduction

*YPEL5* belongs to a novel gene family consisting of five members (*YPEL1–YPEL5*), which is highly conserved among eukaryotic organisms, ranging from fungi to humans (Hosono et al., 2004). Molecular phylogenetic analysis of 100 Yippee-like (YPEL) family members from 68 species reveals that YPEL5 is evolutionarily distinct from the other four members and suggests that YPEL5 is an ortholog of *Drosophila* Yippee (Hosono et al., 2004, 2010). *Drosophila* Yippee was initially identified as an intracellular protein that specifically interacted with the blood protein Hemolin of an insect (moth *Hyalophora cecropia*) (Roxstrom-Lindquist and Faye, 2001). In budding yeast *Saccharomyces cerevisiae*, deletion of *MOH1*, the ortholog of *Drosophila yippee*, enhanced cell viability under various lethal apoptogenic conditions, indicating a pro-apoptotic role of Moh1 protein (Lee et al., 2017). In African green monkey kidney fibroblast COS-7 cells, YPEL5 displays different subcellular localizations during cell cycle progression and positively regulates cell proliferation and growth (Hosono et al., 2010). More recently, it was shown that the *Ypel5* gene is located in a locus linked to a network of interferon-stimulated genes in mice. Further examination shows that YPEL5 is a negative regulator of interferon-β1 production and interacts physically with TBK1/IKBKE kinases in cultured cells (Jeidane et al., 2016). In addition, studies using human colorectal cancer cell lines demonstrate that YPEL5 expression is epigenetically suppressed by the METTL3/YTHDF2 m6A axis (Zhou et al., 2021). Despite accumulating *in vitro* evidence suggests that YPEL5 is multifunctional, its *in vivo* function and mechanism of action are still poorly understood, mainly due to the lack of a vertebrate knockout animal model.

Over the last decades, zebrafish (*Danio rerio*) has emerged as a valuable vertebrate animal model for developmental biology. Compared with other experimental animals, the zebrafish model possesses some unique advantages, including low cost of housing and maintenance, high fecundity, external development, optically transparent embryos, and easy genetic manipulation and screening. Additionally, the major organs and tissues of zebrafish share similar molecular, morphological, and physiological features with their mammalian counterparts. Moreover, the zebrafish and human genomes are well conserved, with ∼70% of human genes having at least one zebrafish ortholog (Howe et al., 2013).

In the present study, we characterize the function of *ypel5* during embryonic development in zebrafish. By loss-of-function assay using a generated CRISPR mutant, we show that *ypel5* deficiency leads to enlarged liver size accompanied by hepatocyte hyperproliferation without significantly increasing cell size. Metabolomic and transcriptomic analyses reveal that hepatic metabolism and function are dysregulated in *ypel5*^−/−^ mutant zebrafish. Furthermore, Hnf4a is identified as a crucial downstream mediator of Ypel5 signaling. The hepatic defects induced by *ypel5* deficiency could be largely rescued by *hnf4a* overexpression. Moreover, peroxisome proliferator-activated receptor α (PPARα) signaling is involved in the regulation of *Hnf4a* by Ypel5. Together, our findings reveal for the first time that Ypel5 is required for normal embryonic liver development and function in vertebrates.

## Results

### Generation of *ypel5^−/−^* mutant zebrafish via the CRISPR/Cas9 system

**Figure 1:**
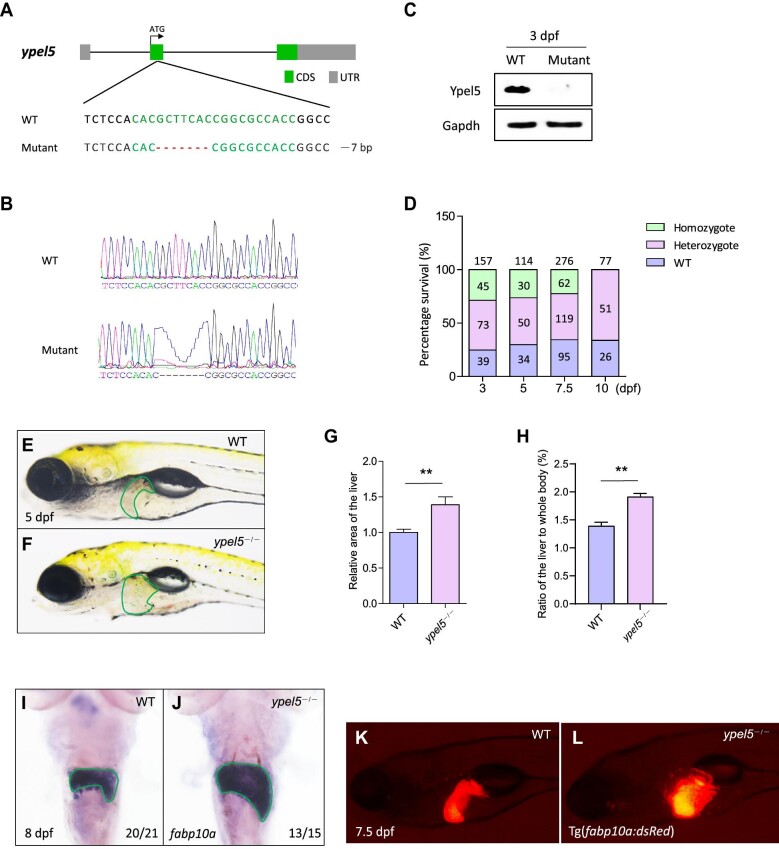
Disruption of the *ypel5* gene causes liver enlargement. (**A**) Genetic inactivation of zebrafish *ypel5* gene based on CRISPR/Cas9. Schematic representation of CRISPR/Cas9 target site at exon 2 as used in this study. The gRNA target site is highlighted in green, and the indel is indicated by a red dash. (**B**) Genotyping of the *ypel5*^−/−^ mutant by Sanger sequencing. (**C**) Western blot analysis of Ypel5 using lysates from wild-type (WT) and *ypel5*^−/−^ mutant embryos at 3 dpf. Gapdh was used as the loading control. (**D**) The survival of zebrafish larvae at different developmental stages. No homozygotes were identified at 10 dpf. (**E–H**) Brightfield images (**E** and **F**), relative liver size (**G**), and the ratio of the liver to whole body (**H**) of WT and *ypel5*^−/−^ mutant zebrafish at 5 dpf. The livers are outlined with green lines. Data shown are mean ± SEM. Student's *t*-test. **P < 0.01. (**I** and **J**) WISH assay of *fabp10a* at 8 dpf. Green lines circle the boundary of the liver. The ratio of embryos with the representative expression pattern is indicated at the right bottom. (**K** and **L**) Representative images of WT and *ypel5*^−/−^/Tg(*fabp10a:dsRed*) zabrafish at 7.5 dpf. The liver was marked by red fluorescence.

To investigate the physiological role of *ypel5*, we generated a *ypel5*-null allele, which harbored a 7-bp deletion in exon 2 (Figure 1A and B). The mutation created a premature stop codon, resulting in the synthesis of a truncated Ypel5 protein lacking 75 C-terminal amino acids. Western blot analysis with the protein extracts from embryos further confirmed the loss of Ypel5 protein in the homozygous mutants (Figure 1C). The *ypel5* homozygous mutants did not exhibit gross morphological defects or developmental delays compared with control siblings during early development (data not shown). However, the *ypel5*^−/−^ larvae could not survive beyond 10 days post fertilization (dpf), as no homozygous mutants were identified at this time point (Figure 1D).

### *ypel5* deficiency leads to hepatomegaly in zebrafish

Although *ypel5*^−/−^ mutants did not display obvious developmental defects in body shape or growth retardation, they exhibited enlarged livers (Figure 1E–H). This prompted us to hypothesize that disruption of *ypel5* expression may affect liver development. We then examined the expression of *fabp10a*, a liver-specific marker, by whole-mount *in situ* hybridization (WISH) assay. The liver size of *ypel5*^−/−^ mutants was significantly increased at 5 dpf (Figure 1E–H), and the enlargement was even more pronounced by 8 dpf (Figure 1I and J). This phenotype was further confirmed on *ypel5*^−/−^/Tg(*fabp10a:dsRed*) larvae where the liver was marked by red fluorescence (Figure 1K and L). However, the bile duct development appeared to be unaffected, as revealed by the Tg(*Tp1bglob:GFP*) line (Supplementary Figure S1A and B). Additionally, the morphologies of other endoderm-derived tissues such as the endocrine pancreas, exocrine pancreas, and intestine were not obviously affected, as determined by the expression of *insulin, trypsin*, and *fabp2*, respectively (Supplementary Figure S1C–H). Together, these results suggest that the loss of Ypel5 affects liver development in zebrafish.

### *ypel5* depletion promotes hepatic cell proliferation

**Figure 2:**
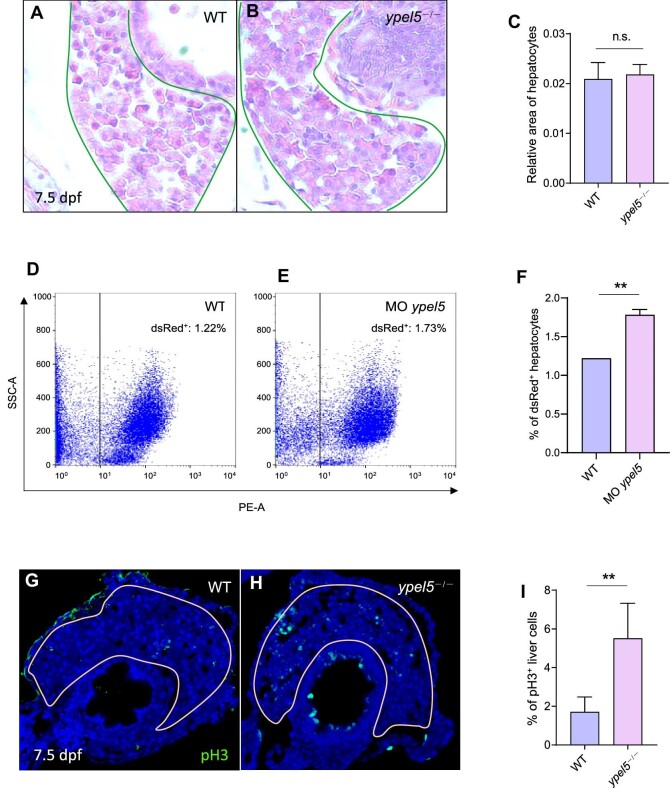
*ypel5* deficiency results in enhanced hepatic cell proliferation. (**A–C**) Representative images of H&E staining (**A** and **B**) and relative hepatocyte size (**C**) of WT and *ypel5*^−/−^ mutant zebrafish at 7.5 dpf. The livers are outlined with green lines. (**D–F**) FACS analysis (**D** and **E**) and quantification (**F**) of dsRed-positive hepatocytes from WT and Tg(*fabp10a:dsRed*) larvae at 7.5 dpf. (**G–I**) Representative images of pH3 staining (green) to label hepatic cell proliferation (**G** and **H**) and quantification of pH3-positive liver cells (**I**) in WT and ypel5^−/−^ mutant zebrafish at 7.5 dpf. The sections were counterstained with DAPI to label the nucleus (blue). White lines circle the boundary of the liver. Data shown are mean ± SEM. Student's *t*-test. n.s., not significant; **P < 0.01.

Hepatomegaly in *ypel5*^−/−^ mutants might result from hepatocyte enlargement and/or hepatic cell hyperproliferation. Hematoxylin and eosin (H&E) histological staining of liver sections did not show significant differences in hepatocyte size between control siblings and *ypel5*-deficient larvae (Figure 2A–C), indicating that *ypel5* deletion has little effect on hepatic cell morphology. In contrast, the number of hepatocytes was significantly increased in the *ypel5* morpholino-injected Tg(*fabp10a:dsRed*) larvae compared with control siblings, as revealed by fluorescence-activated cell sorting analysis (FACS) analysis (Figure 2D–F), suggesting that *ypel5* deletion increased hepatic cell number. To further determine whether hepatocyte proliferation was involved in *ypel5* deficiency-induced hepatomegaly, phosphorylated histone 3 (pH3), a marker of cell proliferation, was examined in liver samples by immunohistochemistry. The percentage of pH3-positive cells was remarkably increased in *ypel5*^−/−^ mutants (Figure 2G–I), suggesting that *ypel5* depletion enhanced hepatocyte proliferation, which may contribute to liver enlargement.

### Hepatic metabolism and function are dysregulated in *ypel5*^−/−^ mutant zebrafish

**Figure 3:**
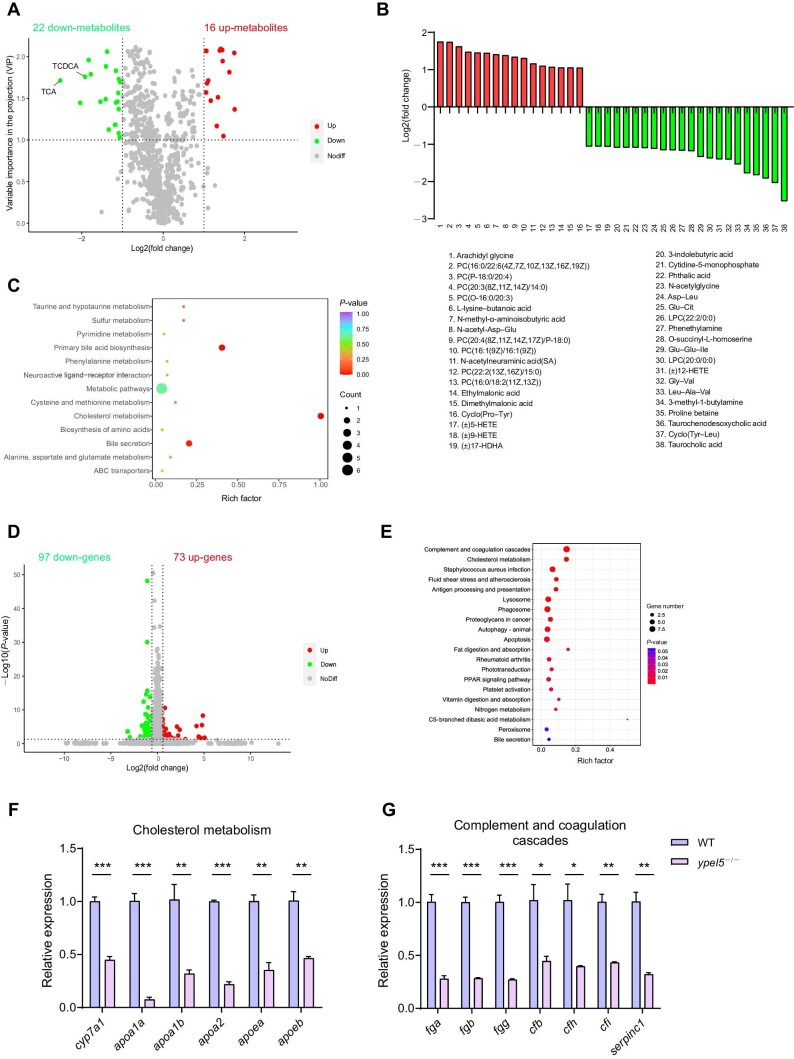
*ypel5* depletion leads to impaired hepatic metabolism and function. (**A–C**) Volcano plot (**A**) and KEGG enrichment (**C**) of differential metabolites between control siblings and *ypel5*^−/−^ mutants at 7.5 dpf. (**B**) The 16 upregulated and 22 downregulated metabolites. (**D** and **E**) Volcano plot (**D**) and KEGG enrichment (**E**) of differentially expressed genes between control siblings and *ypel5*^−/−^ mutants at 3 dpf. (**F** and **G**) qPCR analysis of genes related to cholesterol metabolism and complement and coagulation cascades. Data shown are mean ± SEM. Student's *t*-test. *P < 0.05; **P < 0.01; ***P < 0.001.

The liver is a central metabolic organ. To determine whether *ypel5* deficiency had an impact on hepatic metabolism, a ultrahigh-performance liquid chromatography–electrospray ionization–tandem mass spectrometry (UPLC–ESI–MS/MS)-based, widely targeted metabolomic analysis was applied to reveal the metabolomic profile differences between *ypel5*^−/−^ mutants and control siblings. A total of 818 metabolites were detected in zebrafish larvae extracts at 7.5 dpf. Among them, 22 metabolites were downregulated and 16 metabolites were upregulated in *ypel5*^−/−^ mutants, with variable importance in the projection (VIP) ≥1 and fold change ≥2 (Figure 3A and B; Supplementary Table S1). Of note, taurocholic acid (TCA) and taurochenodesoxycholic acid (TCDCA), which are derived from the catabolism of cholesterol in hepatocytes (de Aguiar et al., 2013), were the most downregulated metabolites. To better understand the biological functions of these differential metabolites, Kyoto Encyclopedia of Genes and Genomes (KEGG) pathway enrichment analysis was employed. Multiple lipid metabolism pathways, such as cholesterol metabolism, primary bile acid biosynthesis, and bile secretion, were affected, and a series of amino acid metabolic pathways, including cysteine and methionine metabolism, were also dysregulated (Figure 3C). Since the liver is a central organ for lipid and amino acid homeostasis, the dysfunction could be a consequence of abnormal hepatic metabolism.

In order to unravel the mechanism behind the altered hepatic metabolism in *ypel5*^−/−^ mutant zebrafish, we conducted RNA sequencing (RNA-seq) with zebrafish larvae at 3 dpf to capture some of the earliest transcriptional changes leading to liver dysfunction. DE-Seq2 was used to determine differentially expressed genes (≥1.5-fold change, *P* < 0.05) between control siblings and *ypel5*^−/−^ mutants. Of the nearly 22693 genes analyzed, 97 genes were downregulated and 73 genes were upregulated in *ypel5*^−/−^ mutants (Figure 3D; Supplementary Table S2). Then, KEGG pathway enrichment analysis was performed to gain insight into the affected biological pathways. Genes associated with cholesterol metabolism were among the top enriched gene sets (Figure 3E), in line with the metabolomic profile of *ypel5*^−/−^ mutants. Additionally, genes associated with complement and coagulation cascades were also highly enriched (Figure 3E). Because the liver is a major organ for the synthesis of coagulation factors and complement, dysregulation of this gene set may contribute to liver dysfunction in *ypel5*^−/−^ mutants.

To further confirm the gene expression differences revealed by RNA-seq, quantitative real-time polymerase chain reaction (qPCR) was performed. The transcriptional level of *cyp7a1*, a rate-limiting enzyme involved in cholesterol metabolism into bile acid, was significantly decreased in *ypel5*^−/−^ mutants, and several genes related to cholesterol transport and metabolism were also downregulated in *ypel5*^−/−^ mutants, including *apoa1a, apoa1b, apoa2, apoea*, and *apoeb* (Figure 3F). Meanwhile, the expression levels of the complement and coagulation cascade genes, *fga, fgb, fgg, cfb, cfh, cfi*, and *serpinc1*, were remarkably reduced in *ypel5*^−/−^ mutants compared to control siblings (Figure 3G). To demonstrate these gene expression changes in the liver, we microinjected *ypel5* morpholino into Tg(*fabp10a:dsRed*) embryos and then sorted the dsRed-positive hepatic cells for qPCR analysis. Consistently, the expression levels of *fga, fgb, fgg, cfb*, and *cfi* were sharply reduced in *ypel5* morphants compared with control embryos (Supplementary Figure S2A). The expression levels of cell cycle-related genes, such as *tp53* and *wee1*, were also decreased in *ypel5* morphants (Supplementary Figure S2B), confirming that *ypel5* deficiency leads to aberrant hepatocyte proliferation. Additionally, we examined the expression of *hnf4a, apoa1b*, and *cfb* by WISH. As expected, their expression was restricted to the liver and downregulated in the mutants (Supplementary Figure S2C).

Taken together, the metabolomic and transcriptomic data suggest that, in addition to controlling hepatocyte proliferation, Ypel5 is also involved in physiological regulation of liver function.

### Hnf4a is a crucial downstream mediator of Ypel5

**Figure 4:**
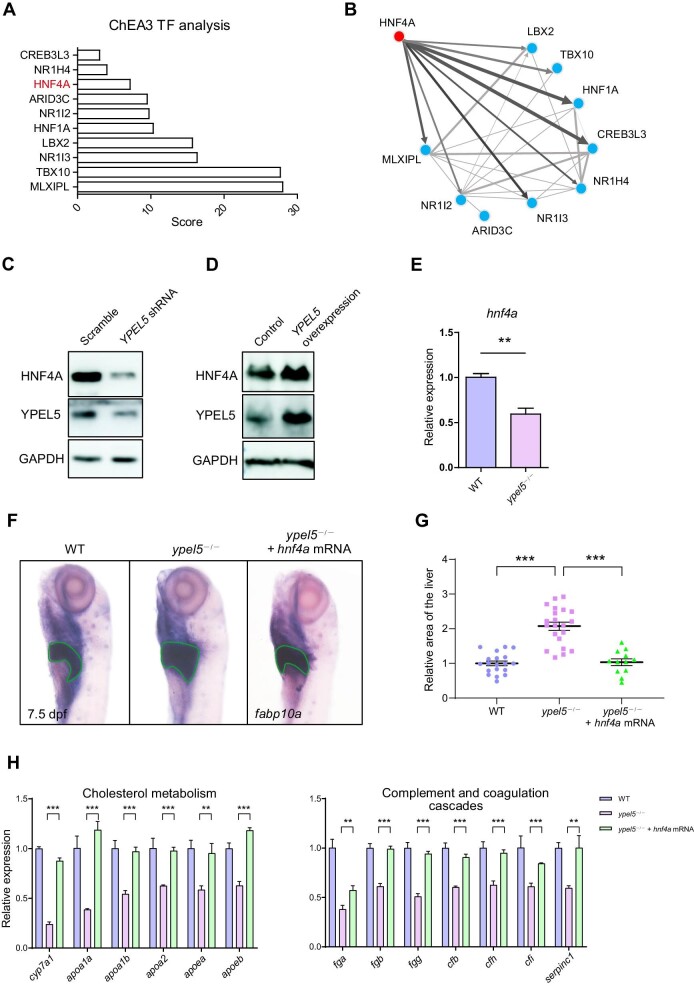
Hnf4a acts as a crucial downstream effector of Ypel5. (**A** and **B**) ChEA3 transcription factor (TF) analysis was performed on differentially expressed genes. The top 10 proteins and their co-regulatory networks are shown. (**C** and **D**) Western blot analysis of HNF4A in Huh-7 cells. (**E**) qPCR analysis of *hnf4a* expression in control siblings and *ypel5*^−/−^ mutants at 3 dpf. (**F** and **G**) WISH assay of *fabp10a* (**F**) and relative liver size (**G**) of zebrafish larvae at 7.5 dpf. Green lines circle the boundary of the liver. (**H**) qPCR analysis of genes related to cholesterol metabolism and complement and coagulation cascades. Data shown are mean ± SEM. Student's *t*-test. **P < 0.01; ***P < 0.001.

In order to identify potential regulators of the *ypel5* deficiency-induced gene expression changes, we performed a transcription factor enrichment analysis, ChEA3 (Keenan et al., 2019). HNF4A was among the top predicted regulators and able to directly regulate other transcription factors (Figure 4A and B). HNF4A is a liver-enriched master regulator that plays a key role in liver development and metabolism (Dubois et al., 2020). As shown in Figure 4C, the protein level of HNF4A was sharply decreased when YPEL5 was knocked down by using short-hairpin RNA (shRNA) in Huh-7 cells. Reciprocally, ectopic expression of YPEL5 induced HNF4A protein level (Figure 4D). The *hnf4a* mRNA level was also significantly reduced in *ypel5*^−/−^ mutants compared with control siblings (Figure 4E; Supplementary Figure S2C). These data suggest that Hnf4a is a downstream effector of Ypel5 and its expression level is positively regulated by Ypel5. To further confirm that Hnf4a mediates the hepatic defects induced by *ypel5* deficiency, we performed a rescue experiment by ectopically expressing *hnf4a* in *ypel5*^−/−^ mutant zebrafish. The hepatomegaly in *ypel5*^−/−^ mutants could be largely ameliorated by *hnf4a* overexpression (Figure 4F and G). Furthermore, the decreased expression levels of genes related to cholesterol metabolism and complement and coagulation cascades were also restored (Figure 4H). Collectively, these findings suggest that Hnf4a is a crucial mediator of *ypel5* deficiency-induced hepatic defects.

### Ypel5 regulates *Hnf4a* expression via the PPARα signaling pathway

**Figure 5:**
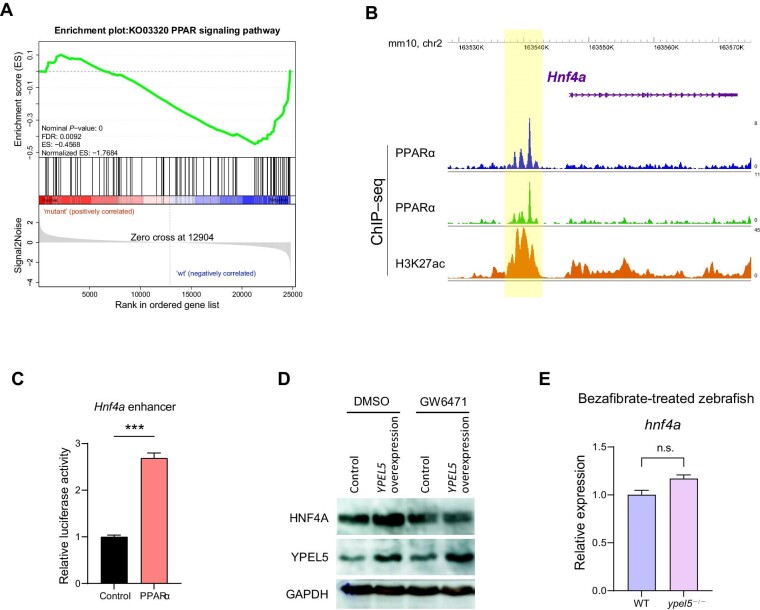
PPARα signaling mediates the regulation of *Hnf4a* by Ypel5. (**A**) GSEA showing the enrichment of the PPAR signaling pathway in *ypel5*^−/−^ mutants. (**B**) Genomic browser view showing ChIP–seq signals of PPARα (GSM864671, GSM1163175) and H3K27ac (GSM2540257) at the *Hnf4a* gene locus in hepatocytes. The peaks are highlighted in yellow. (**C**) Luciferase activity assay in Huh-7 cells transfected with control vectors or PPARα and reporter constructs. The Renilla plasmid was used as an internal control. (**D**) Western blot analysis of Huh-7 cells treated for 48 h with control vehicle or GW6471. (**E**) qPCR analysis of *hnf4a* expression between control siblings and *ypel5*^−/−^ mutants treated for 48 h with bezafibrate. Data shown are mean ± SEM. Student's *t*-test. n.s., not significant; ***P < 0.001.

Gene set enrichment analysis (GSEA) of the RNA-seq data revealed that the PPAR signaling pathway was significantly enriched and downregulated in *ypel5*^−/−^ mutants (Figure 5A). PPARs are lipid-activated transcription factors that belong to the nuclear receptor superfamily. There are three members of this family: PPARα regulates lipid metabolism in the liver, PPARβ/δ promotes fatty acid β-oxidation largely in extrahepatic organs, and PPARγ stores triacylglycerol in adipocytes (Wang et al., 2020). It has been shown that PPARα via HNF4A participated in maintaining metabolic homeostasis in the liver (Contreras et al., 2015). To determine whether PPARα signaling was involved in the regulation of *Hnf4a* expression by Ypel5, we first examined the genome-wide PPARα protein binding sites by applying Cistrome algorithm (Mei et al., 2017) on two PPARα chromatin immunoprecipitation sequencing (ChIP–seq) data sets in hepatic cells (Boergesen et al., 2012; Soltis et al., 2017). There was an intragenic PPARα-bound region ∼4 kb upstream of the *Hnf4a* transcription start site (Figure 5B). This PPARα binding site correlated with histone modification H3K27ac, which is a specific marker for the active enhancers (Creyghton et al., 2010). Then, we cloned this regulatory region into pGL3 luciferase reporter vector and performed transient transfection in Huh-7 cells. As shown in Figure 5C, PPARα overexpression significantly increased luciferase activity. These data suggest that *Hnf4a* may be a direct transcriptional target of PPARα. Furthermore, the elevated HNF4A protein level induced by YPEL5 overexpression was recovered by GW6471 (a potent antagonist of PPARα) treatment in Huh-7 cells (Figure 5D). Bezafibrate, an agonist of PPARα, could fully restore the reduced *hnf4a* expression in *ypel5*^−/−^ mutants (Figure 5E). Taken together, our findings indicate that PPARα signaling mediates the regulation of *Hnf4a* by Ypel5.

## Discussion

YPEL5 is an evolutionarily conserved protein present in a wide variety of eukaryotic organisms, ranging from fungi to vertebrates. To date, its physiological function remains largely unknown. In the current study, we generated a stable *ypel5*^−/−^ mutant zebrafish line by using CRISPR/Cas9 technology and found that the *ypel5* homozygous mutants could not survive beyond 10 dpf. Interestingly, when raised in the sterile egg water supplemented with appropriate antibiotics (Shan et al., 2015), a very small number of *ypel5*^−/−^ larvae could live longer (Supplementary Figure S3A), implying that the compromised immunity might be a reason for the early lethality. Furthermore, *ypel5* depletion led to hepatomegaly accompanied by hepatocyte hyperproliferation without significantly increasing cell size. Consistent with a previous report (Hosono et al., 2004), *ypel5* was ubiquitously expressed during zebrafish embryonic development, including in the liver (Supplementary Figure S4A and B). It is unclear why the *ypel5* depletion-caused predominant tissue-specific phenotypes were generally restricted to the liver. One possible explanation is that the Ypel5 function is compensated by its paralogs in other organs but not in the liver. Supporting this idea, a single-cell transcriptome atlas in developing zebrafish revealed abundant expression of *ypel5* but low or not detectable expression of its paralogs in the liver (Farnsworth et al., 2020; Supplementary Figure S4C). However, we cannot rule out a functional role of Ypel5 in other tissues and organs.

Combined metabolomic and transcriptomic analyses revealed that hepatic metabolism and function were dysregulated in *ypel5*^−/−^ mutant zebrafish. Based on KEGG pathway enrichment analyses of differentially expressed genes and differential metabolites, cholesterol metabolism, one of the important functions of the liver, was identified as the most significantly enriched pathway. TCA and TCDCA are the end-products of cholesterol metabolism in the liver and involved in bile acid biosynthesis. Their levels were sharply reduced in *ypel5*^−/−^ mutants, indicating that cholesterol metabolism, as well as bile acid metabolism, is significantly affected. Accordantly, the transcription level of *cyp7a1*, which catalyzes the first, rate-limiting step in the neutral pathway of bile acid synthesis, was strikingly decreased in *ypel5*^−/−^ mutants. In addition, the mRNA expression levels of genes related to complement and coagulation cascades, which are primarily synthesized in the liver, were also reduced. To overcome the limitations of this study, e.g. not using *ypel5*-depleted hepatocytes for metabolomic and transcriptomic analyses, a mouse line carrying a conditionally null allele of the *Ypel5* gene should be developed and used in future studies.

Zebrafish *ypel5* depletion led to the hepatic phenotype somewhat reminiscent of that seen in *Hnf4a* knockout mice. HNF4A is a principal transcription factor required for liver development and function. It regulates genes specifically implicated in lipid metabolism, glucose metabolism, amino acid metabolism, and blood coagulation (Hayhurst et al., 2001; Inoue et al., 2006). In addition, HNF4A is critical for hepatocyte proliferation. The hepatocyte-specific *Hnf4a* knockout mice displayed severe hepatomegaly and increased hepatocyte proliferation (Hayhurst et al., 2001; Bonzo et al., 2012; Walesky et al., 2013). Here, by performing ChEA3 transcription factor enrichment analysis, we identified Hnf4a as a potential transcription factor that was likely to regulate differentially expressed genes in *ypel5*^−/−^ mutants. We further demonstrated that Hnf4a expression was positively regulated by Ypel5, and overexpression of *hnf4a* could largely rescue the hepatic defects triggered by *ypel5* deficiency. These data indicate that Hnf4a is a key mediator of Ypel5 signaling. Previous reports suggest that YPEL5 is one of the peripheral components of the carboxy-terminal to LisH complex, which has E3 ubiquitin ligase activity (Huffman et al., 2019). However, the regulation of HNF4A by YPEL5 seems not driven by a proteasome-dependent degradation process, because the addition of a proteasome inhibitor did not rescue HNF4A protein levels (Supplementary Figure S5A). Actually, the *hnf4a* mRNA expression level was remarkably decreased in *ypel5*^−/−^ mutants, indicating that the regulation of *hnf4a* expression occurred at the transcriptional level. GSEA of the RNA-seq data revealed that the PPAR signaling pathway was significantly changed in *ypel5*^−/−^ mutants. Further analysis demonstrated that PPARα signaling mediated the regulation of *Hnf4a* by Ypel5 through directly binding to the transcriptional enhancer of the *Hnf4a* gene. It will be of interest to determine how YPEL5 regulates the PPARα signaling pathway during liver development in future studies.

In summary, our results provide the first genetic evidence that Ypel5 is essential for liver development and function, expanding our knowledge to understand the biological functions of the YPEL family and contributing to the development of new targets for the treatment of liver diseases in the future.

## Materials and methods

### Zebrafish

Zebrafish maintenance and staging were performed as described previously (Kimmel et al., 1995). The transgenic lines Tg(*lfabf:dsRed;elaA:EGFP*) (hereafter referred to as Tg(*fabp10a:dsRed*); Korzh et al., 2008) and Tg(*Tp1bglob:GFP*) (Parsons et al., 2009) were used. Relative liver size of zebrafish larvae was measured using ImageJ software. Zebrafish embryos at 24 h post fertilization were treated with 20 μM bezafibrate (Selleck, S4159) or DMSO for 48 h, and then collected for qPCR analysis. The zebrafish facility and study were approved by the Ethics Committee of Ruijin Hospital, Shanghai Jiao Tong University School of Medicine, and the methods were carried out in accordance with the approved guidelines.

### CRISPR/Cas9 mutagenesis

Zebrafish *ypel5* deletion was generated by CRISPR/Cas9-mediated genome editing as described before (Chang et al., 2013). Briefly, guide RNA (gRNA) was designed using an online tool, ZiFiT Targeter software (http://zifit.partners.org/ZiFiT), synthesized *in vitro* with a PCR product-based approach, and purified using mirVana miRNA Isolation Kit (Ambion). Cas9 mRNA was synthesized by *in vitro* transcription using SP6 mMESSAGE mMACHINE Kit (Ambion). Approximately 200 pg of Cas9 mRNA and 50 pg of gRNA were co-injected into 1-cell-stage zebrafish embryos to generate F0 line with mosaic mutation. Further outcrossing of F0 line with wild-type line was performed to generate F1 heterozygous lines. Finally, F2 mutant line with the homozygous mutation was obtained via incrossing of F1 heterozygotes. The *ypel5*^−/−^ mutant line was genotyped by Sanger sequencing of PCR fragments covering the gRNA target site.

### WISH

WISH was performed as described previously (Thisse and Thisse, 2008). Digoxigenin-labeled RNA probes were transcribed with T7, T3, or SP6 polymerase (Ambion). The probes were detected using alkaline phosphatase-coupled anti-digoxigenin Fab fragment antibodies (Roche) with BCIP/NBT staining (Vector Laboratories). The stained embryos were then photographed using a stereomicroscope (Nikon) equipped with a digital camera.

### Histology and pH3 immunostaining

Zebrafish larvae at 7.5 dpf were fixed in 4% paraformaldehyde, dehydrated, embedded in paraffin, cut into 4 μm-thick sections, and then stained with H&E solution according to standard protocols. Relative hepatocyte area was measured using the ImageJ software package.

For pH3 immunostaining, zebrafish larvae at 7.5 dpf were fixed in 4% paraformaldehyde at 4°C overnight, embedded in OCT compound (SAKURA), and cryosectioned into 4-μm slices. After blocked with 10% fetal bovine serum (FBS) for 1 h at room temperature, the sections were incubated with rabbit anti-pH3 antibody (1:100 dilution, Santa Cruz) at 4°C overnight and then with Alexa Fluor 488-conjugated anti-rabbit IgG (Invitrogen) for 1 h at room temperature. The sections were counterstained with DAPI (Vector Labs) to label cell nuclei.

### FACS

Tg(*fabp10a:dsRed*) transgenic larvae at 5 and 7.5 dpf were dissociated into single cells using 0.25% trypsin (Sigma-Aldrich). Single-cell suspension was obtained by centrifugation at 400× *g* for 5 min, washing twice with phosphate-buffered saline, and passing through a 40-μm nylon mesh filter. FACS was performed with FACSAria II (BD Biosciences) to collect homogenous dsRed-positive cells. Data were analyzed with FlowJo 10.8.1 software (FlowJo LLC).

### Targeted metabolomic analysis

Metabolomic analysis was carried out by Wuhan Metware Biotechnology Co., Ltd. Briefly, zebrafish larvae at 7.5 dpf were anesthetized for collection and washed twice with cold HPLC-grade water. The zebrafish were immediately snap-frozen and lysed in 50 μl of pre-cooled 70% methanol/water mixture on ice. Following homogenization, additional 150 μl of pre-cooled 70% methanol/water mixture was supplemented to each tube, followed by incubation on ice for 15 min and centrifugation at 12000 rpm for 10 min. Then, the supernatant was transferred into a new centrifuge tube. After incubating at −20°C for 30 min, the supernatant was centrifuged at 12000 rpm for 15 min at 4°C. The sample extracts were analyzed using an LC–ESI–MS/MS system (UPLC, ExionLC AD; MS, QTRAP® System). The analytical conditions for UPLC were as follows. Column: Waters ACQUITY UPLC HSS T3 C18 (1.8 μm, 2.1 mm × 100 mm); column temperature: 40°C; flow rate: 0.4 ml/min; injection volume: 2 μl; solvent system: water (0.1% formic acid):acetonitrile (0.1% formic acid); gradient program: 95:5 (*v/v*) at 0 min, 10:90 (*v/v*) at 11.0 min, 10:90 (*v/v*) at 12.0 min, 95:5 (*v/v*) at 12.1 min, and 95:5 (*v/v*) at 14.0 min. The metabolites were identified using the Metware database (MWDB). The metabolite abundances were quantified according to their peak areas. Metabolites were considered to be differentially accumulated when VIP ≥ 1 and |log2(fold change)| ≥ 1.

### RNA-seq and data analysis

RNA-seq was carried out by Suzhou Transcriptome Biotechnology Co., Ltd. Briefly, zebrafish larvae at 3 dpf were harvested and lysed in 500 μl TRIzol (Invitrogen), and RNA was extracted following the manufacturer's standard procedures. Libraries were prepared with Illumina TruSeq Stranded mRNA kit and quantified with a Qubit 2.0 Fluorometer (dsDNA HS kit; ThermoFisher), and the size distribution was determined with the Agilent 2100 Bioanalyzer System prior to pooling. RNA-seq was performed on an Illumina HiSeq2000 platform. Sequence reads were aligned to the zebrafish GRCz11 genome assembly with HISAT2 (Kim et al., 2015). Cufflinks v2.2 was used to generate FPKM values, and the differentially expressed genes were determined by R package DE-Seq2 (v 1.34.0) (Trapnell et al., 2013). KEGG pathway enrichment analysis with differentially expressed genes was conducted by R package ClusterProfiler (v 4.2.2). ChEA3 transcription factor enrichment analysis was performed as previously described (Keenan et al., 2019). GSEA was performed by clusterProfiler v3.14.3, using the curated gene set C2 of Molecular Signature Database.

### qPCR

Total RNA was extracted with TRIzol reagent (Invitrogen) according to the manufacturer's recommendations. cDNA was synthesized with the ReverTra Ace-α-™ kit (TOYOBO). qPCR was performed by a LightCycler 480 System (Roche) following the manufacturer's protocols. Relative expression was quantitated using the ΔΔCt method. The primers for qPCR are shown in Supplementary Table S3.

### Plasmid construction and mRNA synthesis

Zebrafish *hnf4a*, human *YPEL5*, and mouse PPARα were cloned into pCS2+ vector. Mouse *Hnf4a* enhancer was cloned into pGL3 promoter vector. The indicated primers for construction are shown in Supplementary Table S1. For shRNA-mediated knockdown, the shRNA (5′-CCGGGTTCGAGATGTGAGCTGCAAACTCGAGTTTGCAGCTCACATCTCGAACTTTTTG-3′) targeting human *YPEL5* mRNA was acquired from MISSION shRNA (Sigma) and cloned into pLKO.1-GFP vector. A scrambled shRNA was used as a control.

Capped mRNAs were synthesized from linearized plasmids using the mMessage mMachine SP6 kit (Invitrogen) and diluted to 60 ng/μl for microinjection.

### Cell culture and transfection

Huh-7 cells were cultured in DMEM (Gibco) supplemented with 10% FBS (Gibco) at 37°C in a humidified atmosphere with 5% CO_2_ and transfected with plasmids using Effectene Transfection Reagent (QIAGEN) according to the manufacturer's protocols. In some experiments, Huh-7 cells were incubated with 20 μM MG132 (MCE, HY-13259) or 10 μM GW6471 (Selleck, S2798) for 24 h or 48 h, respectively, and subjected to western blot analysis.

### Western blot analysis

Western blot analysis was performed using standard methodology with the following antibodies: YPEL5 (Eterlife, EL801477), HNF4A (Cell Signaling Technology, 3113S), and GAPDH (Proteintech, 60004-1).

### ChIP–seq data analysis

PPARα and H3K27ac ChIP–seq data sets in hepatic cells were reanalyzed from previously published work (Boergesen et al., 2012; Goldstein et al., 2017; Soltis et al., 2017). ChIP–seq data were processed by the Cistrome analysis pipeline and loaded into WashU genome browsers for visualization.

### Luciferase activity assay

Huh-7 cells were transfected with the indicated plasmids using Effectene Transfection Reagent (QIAGEN) and harvested after 72 h. Luciferase activities were analyzed using the Dual Luciferase Reporter Assay Kit (Promega) according to the manufacturer's protocols. Luciferase activity was normalized to Renilla activity.

### Statistical analysis

Statistical analysis was performed using GraphPad Prism software. All values are expressed as mean ± SEM except where noted. Differences between two groups were examined using the Student's *t*-test. *P* < 0.05 was considered to be statistically significant.

## Supplementary Material

mjad019_Supplemental_File

## Notes

**Author contributions:** Y.D., X.H., H.C., C.Z., and Y.C. performed experiments and collected data; J. Zhou and H.d.T. provided suggestions on experimental design and data presentation; J. Zhu provided suggestions on experimental design and analyzed data; and H.Y. designed the research, performed experiments, analyzed data, and wrote the manuscript.

## References

[bib1] Boergesen M. , Pedersen T.A., Gross B. et al. (2012). Genome-wide profiling of liver X receptor, retinoid X receptor, and peroxisome proliferator-activated receptor α in mouse liver reveals extensive sharing of binding sites. Mol. Cell. Biol. 32, 852–867. 22158963 10.1128/MCB.06175-11 PMC3272984

[bib2] Bonzo J.A. , Ferry C.H., Matsubara T. et al. (2012). Suppression of hepatocyte proliferation by hepatocyte nuclear factor 4α in adult mice. J. Biol. Chem. 287, 7345–7356. 22241473 10.1074/jbc.M111.334599 PMC3293558

[bib3] Chang N. , Sun C., Gao L. et al. (2013). Genome editing with RNA-guided Cas9 nuclease in zebrafish embryos. Cell Res. 23, 465–472. 23528705 10.1038/cr.2013.45 PMC3616424

[bib4] Contreras A.V. , Rangel-Escareno C., Torres N. et al. (2015). PPARα via HNF4α regulates the expression of genes encoding hepatic amino acid catabolizing enzymes to maintain metabolic homeostasis. Genes Nutr. 10, 452. 25576393 10.1007/s12263-014-0452-0 PMC4288992

[bib5] Creyghton M.P. , Cheng A.W., Welstead G.G. et al. (2010). Histone H3K27ac separates active from poised enhancers and predicts developmental state. Proc. Natl Acad. Sci. USA 107, 21931–21936. 21106759 10.1073/pnas.1016071107 PMC3003124

[bib6] de Aguiar V.T. , Tarling E.J., Edwards P.A. (2013). Pleiotropic roles of bile acids in metabolism. Cell Metab. 17, 657–669. 23602448 10.1016/j.cmet.2013.03.013 PMC3654004

[bib7] Dubois V. , Staels B., Lefebvre P. et al. (2020). Control of cell identity by the nuclear receptor HNF4 in organ pathophysiology. Cells 9, 2185. 32998360 10.3390/cells9102185 PMC7600215

[bib8] Farnsworth D.R. , Saunders L.M., Miller A.C. (2020). A single-cell transcriptome atlas for zebrafish development. Dev. Biol. 459, 100–108. 31782996 10.1016/j.ydbio.2019.11.008 PMC7080588

[bib9] Goldstein I. , Paakinaho V., Baek S. et al. (2017). Synergistic gene expression during the acute phase response is characterized by transcription factor assisted loading. Nat. Commun. 8, 1849. 29185442 10.1038/s41467-017-02055-5 PMC5707366

[bib10] Hayhurst G.P. , Lee Y.H., Lambert G. et al. (2001). Hepatocyte nuclear factor 4α (nuclear receptor 2A1) is essential for maintenance of hepatic gene expression and lipid homeostasis. Mol. Cell. Biol. 21, 1393–1403. 11158324 10.1128/MCB.21.4.1393-1403.2001 PMC99591

[bib11] Hosono K. , Noda S., Shimizu A. et al. (2010). YPEL5 protein of the YPEL gene family is involved in the cell cycle progression by interacting with two distinct proteins RanBPM and RanBP10. Genomics 96, 102–111. 20580816 10.1016/j.ygeno.2010.05.003

[bib12] Hosono K. , Sasaki T., Minoshima S. et al. (2004). Identification and characterization of a novel gene family YPEL in a wide spectrum of eukaryotic species. Gene 340, 31–43. 15556292 10.1016/j.gene.2004.06.014

[bib13] Howe K. , Clark M.D., Torroja C.F. et al. (2013). The zebrafish reference genome sequence and its relationship to the human genome. Nature 496, 498–503. 23594743 10.1038/nature12111 PMC3703927

[bib14] Huffman N. , Palmieri D., Coppola V., (2019). The CTLH complex in cancer cell plasticity. J. Oncol. 2019, 4216750. 31885576 10.1155/2019/4216750 PMC6907057

[bib15] Inoue Y. , Peters L.L., Yim S.H. et al. (2006). Role of hepatocyte nuclear factor 4α in control of blood coagulation factor gene expression. J. Mol. Med. 84, 334–344. 16389552 10.1007/s00109-005-0013-5

[bib16] Jeidane S. , Scott-Boyer M.P., Tremblay N. et al. (2016). Association of a network of interferon-stimulated genes with a locus encoding a negative regulator of non-conventional IKK kinases and IFNB1. Cell Rep. 17, 425–435. 27705791 10.1016/j.celrep.2016.09.009

[bib17] Keenan A.B. , Torre D., Lachmann A. et al. (2019). ChEA3: transcription factor enrichment analysis by orthogonal omics integration. Nucleic Acids Res. 47, W212–W224. 31114921 10.1093/nar/gkz446 PMC6602523

[bib18] Kim D. , Langmead B., Salzberg S.L., (2015). HISAT: a fast spliced aligner with low memory requirements. Nat. Methods 12, 357–360. 25751142 10.1038/nmeth.3317 PMC4655817

[bib19] Kimmel C.B. , Ballard W.W., Kimmel S.R. et al. (1995). Stages of embryonic development of the zebrafish. Dev. Dyn. 203, 253–310. 8589427 10.1002/aja.1002030302

[bib20] Korzh S. , Pan X., Garcia-Lecea M. et al. (2008). Requirement of vasculogenesis and blood circulation in late stages of liver growth in zebrafish. BMC Dev. Biol. 8, 84. 18796162 10.1186/1471-213X-8-84 PMC2564926

[bib21] Lee J.Y. , Jun D.Y., Park J.E. et al. (2017). Pro-apoptotic role of the human YPEL5 gene identified by functional complementation of a yeast moh1Δ mutation. J. Microbiol. Biotechnol. 27, 633–643. 28173693 10.4014/jmb.1610.10045

[bib22] Mei S. , Qin Q., Wu Q. et al. (2017). Cistrome data browser: a data portal for ChIP–seq and chromatin accessibility data in human and mouse. Nucleic Acids Res. 45, D658–D662. 27789702 10.1093/nar/gkw983 PMC5210658

[bib23] Parsons M.J. , Pisharath H., Yusuff S. et al. (2009). Notch-responsive cells initiate the secondary transition in larval zebrafish pancreas. Mech. Dev. 126, 898–912. 19595765 10.1016/j.mod.2009.07.002 PMC3640481

[bib24] Roxstrom-Lindquist K. , Faye I. (2001). The Drosophila gene Yippee reveals a novel family of putative zinc binding proteins highly conserved among eukaryotes. Insect Mol. Biol. 10, 77–86. 11240639 10.1046/j.1365-2583.2001.00239.x

[bib25] Shan Y. , Fang C., Cheng C. et al. (2015). Immersion infection of germ-free zebrafish with Listeria monocytogenes induces transient expression of innate immune response genes. Front. Microbiol. 6, 373. 25972853 10.3389/fmicb.2015.00373 PMC4413826

[bib26] Soltis A.R. , Motola S., Vernia S. et al. (2017). Hyper- and hypo-nutrition studies of the hepatic transcriptome and epigenome suggest that PPARα regulates anaerobic glycolysis. Sci. Rep. 7, 174. 28282965 10.1038/s41598-017-00267-9 PMC5428070

[bib27] Thisse C. , Thisse B. (2008). High-resolution in situ hybridization to whole-mount zebrafish embryos. Nat. Protoc. 3, 59–69. 18193022 10.1038/nprot.2007.514

[bib28] Trapnell C. , Hendrickson D.G., Sauvageau M. et al. (2013). Differential analysis of gene regulation at transcript resolution with RNA-seq. Nat. Biotechnol. 31, 46–53. 23222703 10.1038/nbt.2450 PMC3869392

[bib29] Walesky C. , Gunewardena S., Terwilliger E.F. et al. (2013). Hepatocyte-specific deletion of hepatocyte nuclear factor-4α in adult mice results in increased hepatocyte proliferation. Am. J. Physiol. Gastrointest. Liver Physiol. 304, G26–G37. 23104559 10.1152/ajpgi.00064.2012 PMC3543634

[bib30] Wang Y. , Nakajima T., Gonzalez F.J. et al. (2020). PPARs as metabolic regulators in the liver: lessons from liver-specific PPAR-null mice. Int. J. Mol. Sci. 21, 2061. 32192216 10.3390/ijms21062061 PMC7139552

[bib31] Zhou D. , Tang W., Xu Y. et al. (2021). METTL3/YTHDF2 m6A axis accelerates colorectal carcinogenesis through epigenetically suppressing YPEL5. Mol. Oncol. 15, 2172–2184. 33411363 10.1002/1878-0261.12898 PMC8333777

