# Supplementary material for: Ypel5 regulates liver development and function in zebrafish
Source: J Mol Cell Biol. 2023 Mar 22;15(3):mjad019. doi: 10.1093/jmcb/mjad019 (PMC10588938; doi:10.1093/jmcb/mjad019)
Supplement: mjad019_Supplemental_File [file mjad019_supplemental_file.pdf]

Supporting Information for：

Ypel5 regulates liver development and function in zebrafish

Yun Deng[1, 2, †], Xiao Han[1, 2, †], Huiqiao Chen[3], Chaoxian Zhao[1, 2], Yi Chen[1], Jun Zhou[1, 2], Hugues de The[2, 4], Jun Zhu[2, 4, *] and Hao Yuan[1, 2, *]

1. Shanghai Institute of Hematology, State Key Laboratory of Medical Genomics, National Research Center for Translational Medicine at Shanghai, Ruijin Hospital, Shanghai Jiao Tong University School of Medicine, Shanghai 200025, China.

2. CNRS-LIA Hematology and Cancer, Sino-French Research Center for Life Sciences and Genomics, Ruijin Hospital, Shanghai Jiao Tong University School of Medicine, Shanghai 200025, China.

3. Department of Hematology, Sir Run Run Shaw Hospital, Zhejiang University School of Medicine, Hangzhou 310011, China.

4. Université de Paris 7/INSERM/CNRS UMR 944/7212, Equipe Labellisée Ligue Nationale Contre le Cancer, Hôpital St. Louis, Paris 75010, France.

*Correspondence to: Dr. Jun Zhu (zhuj1966@yahoo.com or jun.zhu@paris7.jussieu.fr) or Dr. Hao Yuan (hyuan@sibs.ac.cn).

†Y. D and X. H contributed equally to this study.

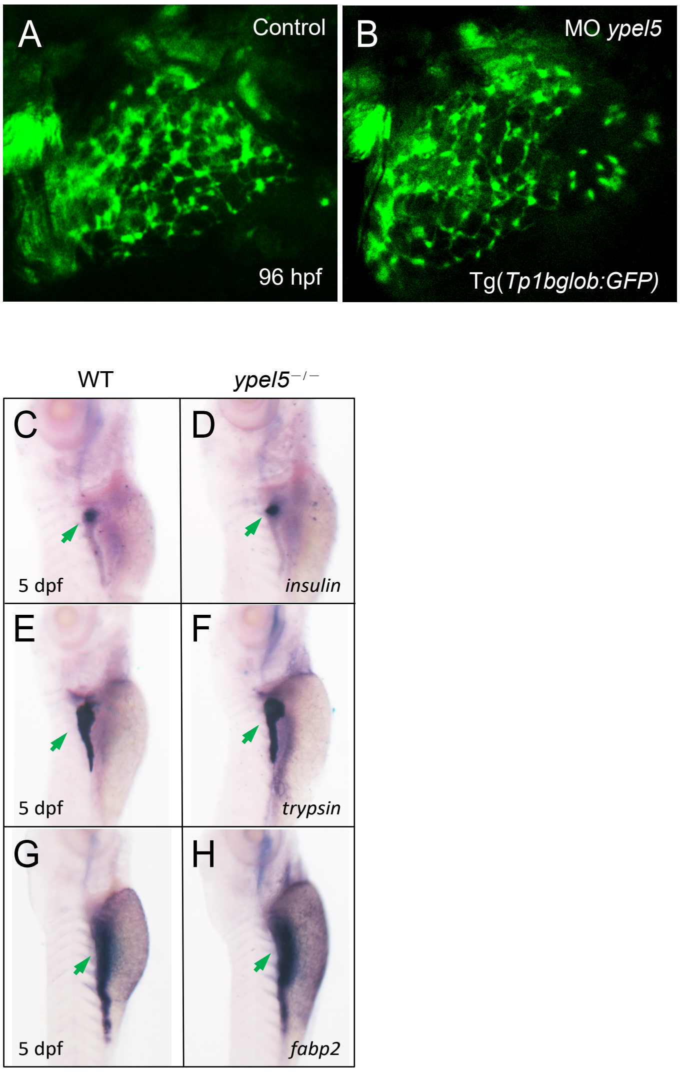

Supplementary Figure S1. The development of bile duct and the morphology of endocrine pancreas, exocrine pancreas, and intestine are not significantly affected in *ypel5*$^{-/-}$ mutant. (A-B) Representative images from control sibling and the *ypel5* morpholino-injected Tg(*Tp1bglob:GFP*) at 96 hpf. (C-H) WISH assay of *insulin*, *trypsin*, and *fabp2* at 5 dpf. Green arrows indicate endocrine pancreas, exocrine pancreas, and intestine, respectively.

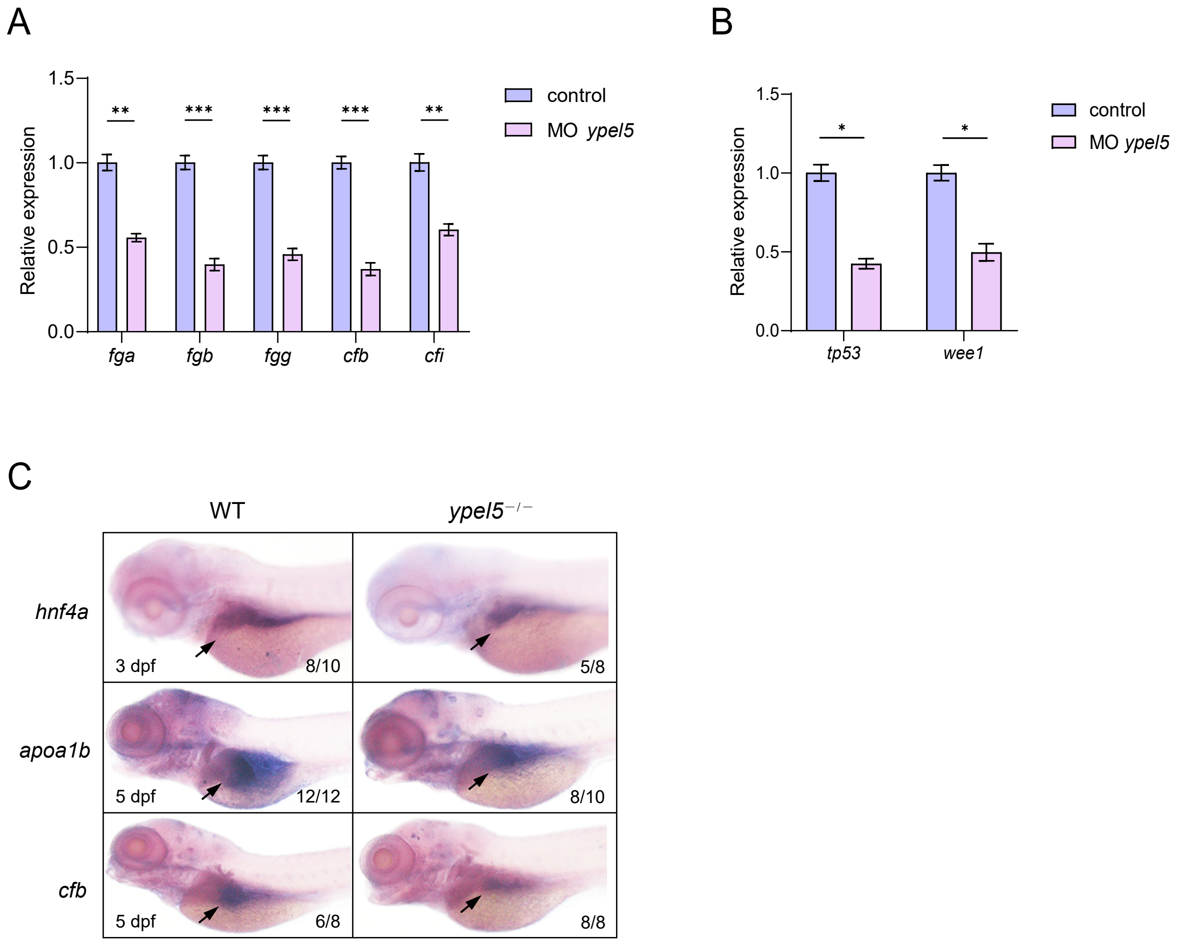

Supplementary Figure S2. *ypel5* deficiency results in gene expression changes in the liver. (A-B) Quantitative PCR analysis was performed in dsRed-positive hepatic cells sorted from Tg(*fabp10a:dsRed*) larvae at 5 dpf. Data shown are the means ± SEM. Statistical significance was calculated using the Student's *t*-test. *, $p<0.05$; **, $p<0.01$; ***, $p<0.001$. (C) WISH assay of *hnf4a*, *apoa1b* and *cfb* at 3 and 5 dpf, respectively.

A

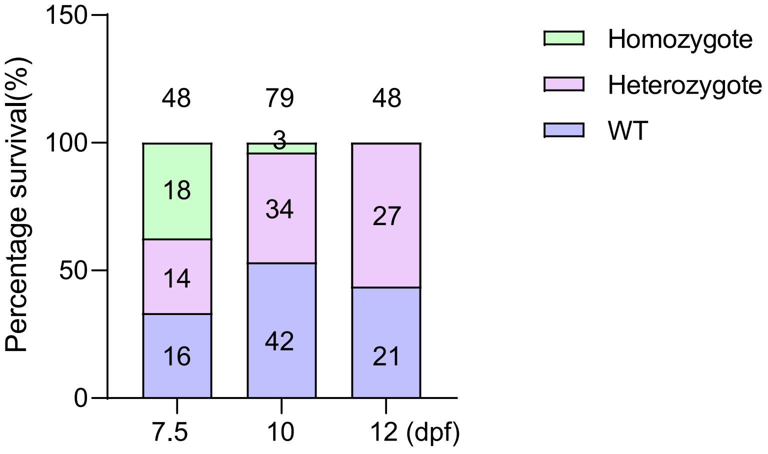

Supplementary Figure S3. Antibiotics treatment allows the *ypel5*$^{-/-}$ mutant to live longer. (A) The survival of embryos raised in sterile egg water supplemented with ampicillin and kanamycin. Of note, a very small number of *ypel5*$^{-/-}$ larvae could be detected at 10 dpf (3/79), which was never seen in larvae raised in normal egg water.

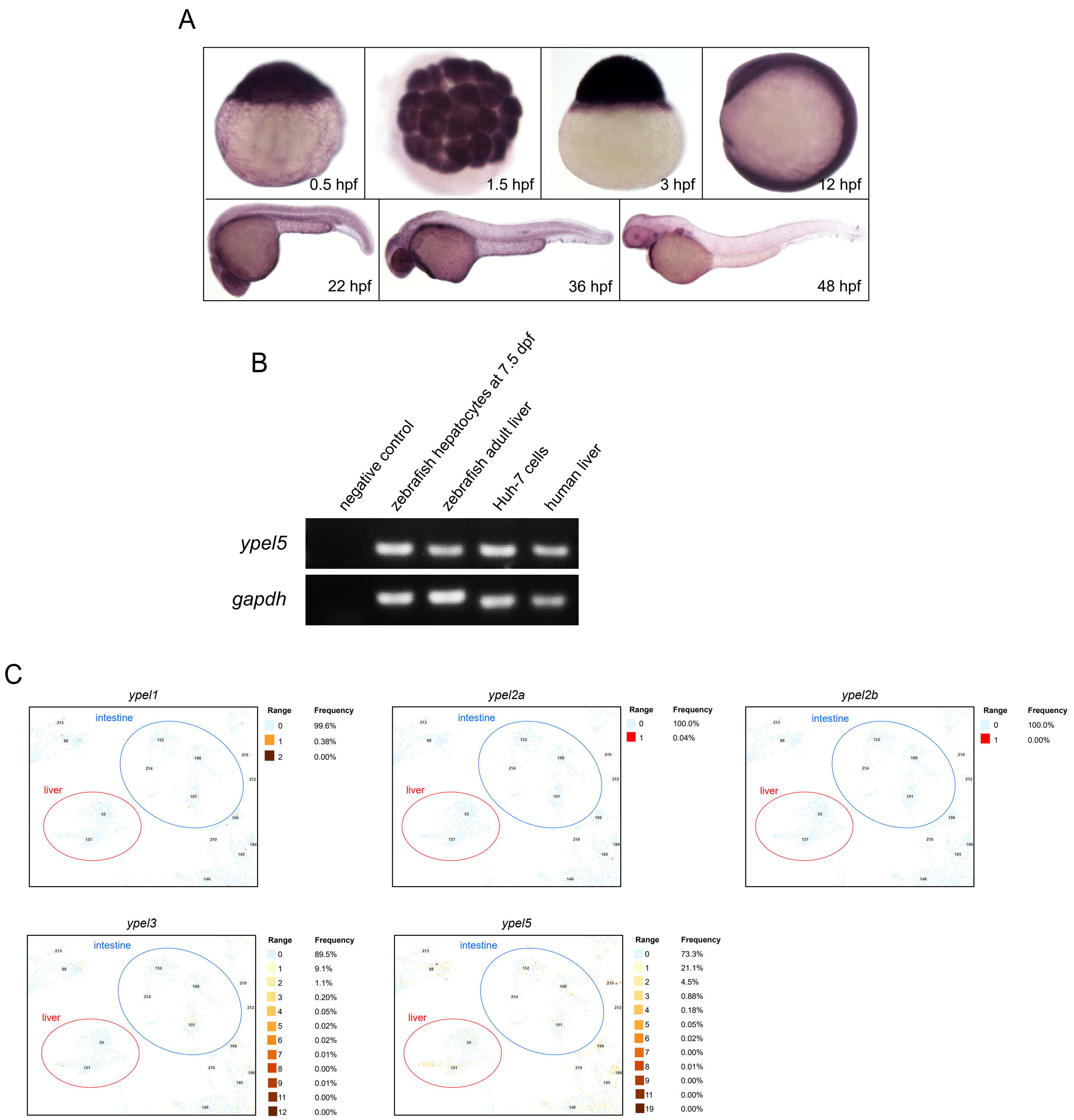

Supplementary Figure S4. The expression profile of *ypel5*. (A) WISH assay of *ypel5* during early zebrafish embryonic development. (B) Semi-quantitative RT-PCR analysis of *ypel5* transcripts. Total RNA were isolated from different samples, including hepatocytes sorted from Tg(*fabp10a:dsRed*) at 7.5 dpf, zebrafish adult liver, human hepatocellular carcinoma Huh-7 cells, and human liver. The *gapdh* expression was analyzed as an endogenous control. Sterile water was used as the internal negative control. (C) single-cell RNA-sequencing atlas of developing zebrafish embryos during organogenesis.

A

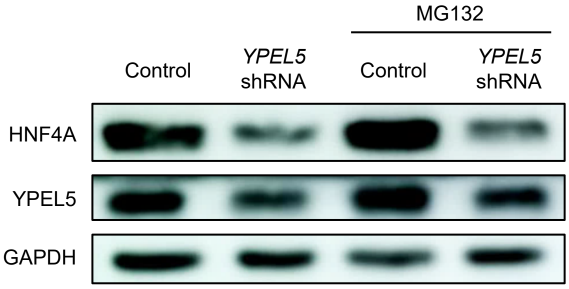

Supplementary Figure S5. Regulation by YPEL5 of HNF4A is not driven by a proteasome-dependent degradation process. (A) Western blot analysis of HNF4A in Huh-7 cells treated with or without MG132.

Supplementary Table S1. The downregulated and upregulated metabolites.

Index	Formula	Compounds	Class I	Class II	WT-rep1	WT-rep2	WT-rep3	*ypel5* mutant-rep1	*ypel5* mutant-rep2	*ypel5* mutant-rep3	VIP	p_value	Fold_Char	Log2FC	Type
MEDP1131	C22H43NO3	Arachidyl glycine	Alcohol and amines	Amines	45312	83436	51536	84511	436350	86875	1.367886	0.346905	3.370992	1.753173	up
MADP0468	C46H80NO8P	PC(16:0/22:6(4Z,7Z,10Z,13Z,16Z,19Z)	GP	PC	309320	581640	481550	1890300	1389200	1318900	2.045518	0.015	3.350358	1.744315	up
MADP0474	C46H84NO7P	PC(P-18:0/20:4)	GP	PC-P	31626	84255	92059	263810	229730	147170	1.814879	0.033489	3.081225	1.623504	up
MADP0449	C42H78NO8P	PC(20:3(8Z,11Z,14Z)/14:0)	GP	PC	19882	103000	107820	51587	435090	155930	1.046689	0.353139	2.785442	1.477906	up
MADP0477	C44H84NO7P	PC(O-16:0/20:3)	GP	PC-O	124020	133960	243690	562170	442890	374260	1.947763	0.015305	2.749457	1.459147	up
MEDP1968	C10H22N2O4	L-Lysine-Butanoic Acid	Amino acid and Its metabolomics	Amino acid derivatives	31054000	31884000	24145000	88898000	87701000	61366000	2.08185	0.023928	2.732623	1.450286	up
MEDP1177	C5H11NO2	N-Methyl-α-aminoisobutyric acid	Amino acid and Its metabolomics	Amino acid derivatives	28159000	31543000	23710000	80337000	82696000	58874000	2.093324	0.018976	2.660373	1.411628	up
MEDN0561	C11H16N2O8	N-Acetyl-Asp-Glu	Amino acid and Its metabolomics	Small Peptide	480890	528630	402160	1312800	1428200	952180	2.076625	0.027517	2.616159	1.38745	up
MADP0436	C46H84NO7P	PC(20:4(8Z,11Z,14Z,17Z)/P-18:0)	GP	PC	23446	78584	107400	272660	136660	122410	1.513168	0.140324	2.538939	1.344226	up
MADP0451	C40H76NO8P	PC(16:1(9Z)/16:1(9Z))	GP	PC	18074	82124	50342	40570	230350	103590	1.167988	0.311958	2.487777	1.314857	up
MADN0215	C11H19NO9	N-Acetylneuraminic Acid(SA)	Amino acid and Its metabolomics	Amino acid derivatives	4511.6	1826.7	3647.1	9624.1	9117.5	3676.8	1.472503	0.149116	2.245118	1.166791	up
MADP0447	C45H86NO8P	PC(22:2(13Z,16Z)/15:0)	GP	PC	25630	35216	64914	88336	67621	114790	1.713137	0.056643	2.152886	1.106272	up
MADP0469	C42H80NO8P	PC(16:0/18:2(11Z,13Z))	GP	PC	44224	53043	124980	128630	171250	168490	1.681455	0.064746	2.10743	1.075485	up
MEDN0413	C5H8O4	Ethylmalonic acid	Organic acid And Its derivatives	Organic acid And Its derivatives	45333	32296	41330	77820	90349	79303	2.070609	0.001491	2.080313	1.056801	up
MEDN1533	C5H8O4	Dimethylmalonic acid	Organic acid And Its derivatives	Organic acid And Its derivatives	45333	32296	41330	77820	90349	79303	2.070609	0.001491	2.080313	1.056801	up
MADN0438	C14H16N2O3	cyclo(pro-tyr)	Amino acid and Its metabolomics	Small Peptide	11917	23463	15812	21518	29642	55141	1.570804	0.204394	2.076516	1.054165	up
MEDN0760	C20H32O3	(±)5-HETE	FA	Oxidized lipids	103200	113850	109410	32731	91879	31881	1.697124	0.100699	0.479357	-1.06083	down
MEDN0763	C20H32O3	(±)9-HETE	FA	Oxidized lipids	103200	113850	109410	32731	91879	31881	1.697124	0.100699	0.479357	-1.06083	down
MEDN0754	C22H32O3	(±)17-HDHA	FA	Oxidized lipids	13112	65017	54040	13764	33698	15593	1.030633	0.280497	0.477079	-1.0677	down
MEDN0260	C12H13NO2	3-Indolebutyric Acid	Heterocyclic compounds	Indole and Its derivatives	7927.3	10239	6660	6223.3	2495.5	2941.2	1.731559	0.050181	0.469663	-1.0903	down
MADP0066	C9H14N3O8P	Cytidine-5-Monophosphate	Nucleotide And Its metabolomics	Nucleotide and Its metabolomics	107700	66197	47208	73585	21156	9077.1	1.370561	0.217207	0.469542	-1.09067	down
MADN0270	C8H6O4	Phthalic Acid	Benzene and substituted derivatives	Phenolic acids	1353500	366750	325700	410160	286880	263090	1.080257	0.394122	0.469283	-1.09147	down
MADN0118	C4H7NO3	N-Acetylglycine	Amino acid and Its metabolomics	Amino acid derivatives	32670	16332	20796	19491	7234.9	5786	1.564155	0.130754	0.4658	-1.10222	down
MADP0424	C10H18N2O5	Asp-Leu	Amino acid and Its metabolomics	Small Peptide	31878	93224	87946	36337	39538	22063	1.460491	0.183281	0.459699	-1.12124	down
MADP0408	C11H20N4O6	Glu-Cit	Amino acid and Its metabolomics	Small Peptide	20025	65764	57818	19908	19819	24540	1.449784	0.200462	0.44752	-1.15998	down
MEDP1689	C30H58NO7P	LPC(22:2/0:0)	GP	LPC	58894	101480	134940	43412	43184	45499	1.832371	0.13181	0.447304	-1.16067	down
MEDP2058	C8H11N	Phenethylamine	Alcohol and amines	Amines	1378100	659300	2971000	928810	246410	1047300	1.1823	0.306414	0.443758	-1.17215	down
MEDP0876	C8H13NO6	O-Succinyl-L-Homoserine	Amino acid and Its metabolomics	Amino acid derivatives	2315.5	3909.5	2926.5	1087.9	2930	9	1.182419	0.174086	0.440026	-1.18434	down
MEDP1671	C16H27N3O8	Glu-Glu-Ile	Amino acid and Its metabolomics	Small Peptide	81185	531870	199310	42075	210790	68938	1.124225	0.352255	0.396131	-1.33595	down
MEDP1690	C28H58NO7P	LPC(20:0/0:0)	GP	LPC	120600	94011	118230	49356	47727	31025	2.061952	0.004083	0.384892	-1.37747	down
MEDN0751	C20H32O3	(±)12-HETE	FA	Oxidized lipids	255830	215390	255480	57137	152170	65092	1.884709	0.024184	0.377596	-1.40508	down
MADN0262	C7H14N2O3	Gly-Val	Amino acid and Its metabolomics	Small Peptide	15103	47941	16100	5847.8	15593	8308.8	1.490613	0.263136	0.375892	-1.41161	down
MEDP1668	C14H27N3O4	Leu-Ala-Val	Amino acid and Its metabolomics	Small Peptide	23386	109750	50056	13627	32300	17092	1.460454	0.254343	0.344005	-1.5395	down
MADP0114	C5H13N	3-Methyl-1-butylamine	Alcohols and amines	Amines	106470	142350	47665	20087	41721	24659	1.789575	0.119665	0.29164	-1.77774	down
MADP0208	C7H13NO2	Proline betaine	Organic acid And Its derivatives	Organic acid And Its derivatives	516520	224020	239700	77377	93052	105330	1.959785	0.131001	0.281318	-1.82973	down
MEDN0106	C26H45NO6S	Taurochenodesoxycholic Acid	Bile acids	Bile acids	340220	841640	376290	56526	221030	135180	1.759479	0.132603	0.264888	-1.91654	down
MEDP1931	C15H20N2O2	Cyclo(Tyr-Leu)	Amino acid and Its metabolomics	Small Peptide	5369.1	33492	7464.8	2208	4544.7	4539.1	1.447049	0.325538	0.243747	-2.03654	down
MEDN0105	C26H45NO7S	Taurocholic acid	Bile acids	Bile acids	171020	659800	215490	15997	115040	50750	1.714511	0.202856	0.173741	-2.52499	down

Supplementary Table S2. The downregulated and upregulated genes.

Gene ID	Gene Symbol	WT-rep1	WT-rep2	*ypel5* mutant-rep1	*ypel5* mutant-rep2	log2(fc)	PValue
ENSDARG00000056511	arr3a	0.09	0	2.82	0.21	5.0732	0.018398
ENSDARG00000087651	MGC174155	0.47	5.17	6.95	159.5	4.8832	5.07E-09
ENSDARG00000089750	si:dkey-26g8.5	0	0.55	0.28	14.82	4.779	3.31E-06
ENSDARG00000094559	zgc:174855	0.28	1.1	0.84	33.43	4.6342	0.020066
ENSDARG00000100833	LOC100536033	0.38	1.87	1.13	47.21	4.4252	0.008301
ENSDARG00000095072	si:dkey-26g8.4	0.66	4.84	5.16	95.89	4.1995	6.08E-06
ENSDARG00000038728	ch25hl2	0.19	0	1.41	0.11	3	0.045628
ENSDARG00000038147	hbbe3	1.42	5.06	4.13	30.07	2.3999	7.57E-05
ENSDARG00000045677	opn1sw1	0.47	0.11	2.72	0	2.2295	0.003923
ENSDARG00000074306	wu:fa26c03	1.42	12.55	2.07	57.51	2.0925	8.79E-06
ENSDARG00000078193	si:ch211-67e16.3	0.47	0.11	1.88	0.21	1.8494	0.037026
ENSDARG00000035422	ccn1l1	1.33	0.99	5.54	1.05	1.5062	0.017379
ENSDARG00000002193	rho	0.57	0.55	3	0.11	1.4734	0.034498
ENSDARG00000041589	adprhl1	0.09	1.1	0.09	3.05	1.3998	0.015508
ENSDARG00000089806	si:dkey-239j18.3	7.41	38.31	5.16	105.03	1.2691	0.001431
ENSDARG00000025797	abhd2a	0.09	0.88	0	2.31	1.2518	0.02352
ENSDARG00000100576	LOC100334521	1.52	0.44	3.47	1.05	1.2055	0.014672
ENSDARG00000023656	he1.1	9.59	52.07	5.44	133.53	1.1724	0.001769
ENSDARG00000019122	he1.2	5.32	21.8	2.07	57.62	1.1381	0.012902
ENSDARG00000104919	si:ch211-153b23.	2.18	3.96	1.22	11.04	0.9976	0.032941
ENSDARG00000045956	kcnk10b	0	1.21	0.19	2.21	0.988	0.021062
ENSDARG00000002696	gnb3b	0.57	0.22	1.5	0	0.925	0.029418
ENSDARG00000086512	prodhb	11.87	5.06	19.81	12.09	0.914	4.30E-05
ENSDARG00000067701	myoz3a	9.21	7.93	18.78	13.46	0.9115	0.012455
ENSDARG00000004049	marcksa	3.23	8.92	6.1	16.3	0.8825	0.040924
ENSDARG00000054680	chrna9a	1.9	0.55	3.1	1.37	0.8675	0.026817
ENSDARG00000042529	gnat2	0.57	0.11	1.22	0	0.8433	0.028765
ENSDARG00000000380	pde6a	0.95	0.11	1.69	0.21	0.8419	0.015361
ENSDARG00000034457	si:ch211-163l21.	1.71	0.55	3	1.05	0.8416	0.024436
ENSDARG00000057859	hoxd10a	11.3	12.88	28.54	14.51	0.8322	0.007121
ENSDARG00000056023	hoxb9a	33.43	34.68	67.68	52.15	0.8151	0.001373
ENSDARG00000099702	ahrra	2.75	9.69	5.07	16.72	0.8087	0.006374
ENSDARG00000101195	cyp1c1	39.41	119.11	60.64	217.01	0.8086	2.67E-11
ENSDARG00000029830	myf6	4.08	1.98	6.48	4.1	0.8039	0.018766
ENSDARG00000021265	mybpc2b	190.58	202.66	362.46	303.75	0.7606	4.46E-05
ENSDARG00000036722	slc9a3.2	3.89	1.54	5.07	4.1	0.756	0.03829
ENSDARG00000075299	prxl2c	2.94	2.97	7.7	2.21	0.7457	0.025437
ENSDARG00000053315	tmprss3a	17.95	14.64	35.77	18.71	0.7413	7.59E-05
ENSDARG00000073978	crabp2a	40.64	39.96	72.75	59.51	0.7145	0.00071
ENSDARG00000045071	chad	12.82	14.2	26	18.29	0.713	0.022158
ENSDARG00000060330	si:ch211-284e13.	1.33	5.72	2.53	8.94	0.7022	0.019611
ENSDARG00000034423	sncga	57.16	65.39	110.49	87.37	0.6911	0.001812
ENSDARG00000044512	ca4c	4.27	2.42	6.57	4.21	0.6883	0.026288
ENSDARG00000095147	krt96	12.44	6.83	19.71	10.83	0.6643	6.61E-05
ENSDARG00000087784	si:dkeyp-110a12.	18.8	13.76	30.51	21.03	0.6626	0.000257
ENSDARG00000077996	LOC100007192	1.8	7.05	3.47	10.51	0.6596	0.023578
ENSDARG00000011579	hoxb10a	16.52	13.87	29.66	18.29	0.6579	0.000618
ENSDARG00000100958	LOC103909734	1.9	8.81	4.04	12.83	0.6555	0.006263
ENSDARG00000086724	im:7151449	7.31	4.73	11.36	7.46	0.6444	0.008147
ENSDARG00000042172	c7a	24.97	18.71	36.24	31.96	0.6428	0.001138
ENSDARG00000114407	zgc:77614	0.66	0	1.03	0	0.6421	0.007124
ENSDARG00000100968	si:ch211-1a19.3	7.5	5.17	12.39	7.36	0.6404	0.00551
ENSDARG00000101789	cyp1c2	6.65	13.87	7.42	24.5	0.6374	0.022444
ENSDARG00000098823	pdzd4	3.99	12.33	6.01	19.35	0.6359	0.006032
ENSDARG00000062449	xpr1a	4.18	12.44	7.51	18.19	0.6288	0.028814
ENSDARG00000059387	fgf7	0.47	4.29	1.78	5.57	0.6268	0.023403
ENSDARG00000062788	irg1l	7.22	17.06	5.63	31.86	0.6267	0.000638
ENSDARG00000092809	hoxc9a	15.95	12.99	24.88	19.77	0.6256	0.004324
ENSDARG00000099265	slc7a5	2.47	8.48	3.94	12.72	0.6055	0.015754
ENSDARG00000015559	fstl1a	22.51	19.04	37.93	25.23	0.6042	0.000288
ENSDARG00000102879	myt1b	18.9	56.58	38.11	76.54	0.6031	0.001465
ENSDARG00000068247	luzp2	3.7	13.1	7.32	18.19	0.6026	0.013077
ENSDARG00000097487	pfn4	1.8	0.22	2.53	0.53	0.5992	0.004125
ENSDARG00000068934	cyp1b1	3.99	18.05	5.73	27.55	0.5945	5.37E-06
ENSDARG00000077906	rnf165a	5.41	20.04	6.95	31.44	0.5931	1.55E-05
ENSDARG00000103862	hoxa3a; hoxa4a	6.93	21.14	10.8	31.54	0.593	0.001283
ENSDARG00000054941	ldlrad4b	2.94	7.38	2.72	12.83	0.5915	0.018233

ENSDARG00000030650	r3hdml	3.13	1.65	4.88	2.31	0.589	0.023281
ENSDARG00000056092	si:dkey-12h9.6	3.89	12.33	6.1	18.29	0.5885	0.008495
ENSDARG00000088136	pcdh2aa15	12.63	47.45	22.81	67.39	0.5862	1.24E-06
ENSDARG00000101641	trpm2	0.47	3.3	1.03	4.63	0.5862	0.027001
ENSDARG00000077349	sorcs3b	0.38	4.29	2.07	4.94	0.586	0.038754
ENSDARG00000040252	atp1a1a.5	40.17	28.07	64.4	37.96	0.585	7.44E-08
ENSDARG00000022971	epha6	4.27	10.24	1.97	7.68	-0.5884	0.047838
ENSDARG00000115717	pigbos1	19.37	13.21	13.42	8.2	-0.5916	0.000582
ENSDARG00000101393	si:dkey-31g6.6	9.97	26.2	4.22	19.77	-0.5924	0.000754
ENSDARG00000063412	cers1	1.8	7.49	1.22	4.94	-0.5927	0.023591
ENSDARG00000038153	lgals2b	121.36	82.67	66.28	68.87	-0.5942	8.14E-05
ENSDARG00000039173	ctslb	0.85	3.41	0.19	2.63	-0.5952	0.032636
ENSDARG00000020952	naaladl1	13.86	13.65	10.51	7.68	-0.5968	0.038589
ENSDARG00000095002	tnnc2.1	4.75	2.31	3.29	1.37	-0.5993	0.014284
ENSDARG00000055723	hsp70l	7.6	2.64	3.38	3.36	-0.6034	0.032785
ENSDARG00000058734	prdx1	87.84	77.28	46.84	61.82	-0.6037	0.03774
ENSDARG00000077112	si:ch211-180f4.1	1.52	10.13	1.88	5.78	-0.6049	0.008136
ENSDARG00000070078	abcb11b	3.13	5.61	0.38	5.36	-0.6066	0.041755
ENSDARG00000030215	matn1	176.62	451.12	65.53	342.33	-0.6221	0.00802
ENSDARG00000068460	otog	3.61	15.3	1.03	11.25	-0.6228	5.18E-05
ENSDARG00000104129	si:dkey-23n7.10	2.75	1.1	2.07	0.42	-0.6287	0.014988
ENSDARG00000042428	gstt1a	80.24	79.15	47.41	54.88	-0.6399	0.022192
ENSDARG00000099032	LOC108190739	13.96	7.27	6.38	7.15	-0.6499	0.034327
ENSDARG00000061120	slc43a2b	48.43	110.85	31.54	69.92	-0.6507	0.001191
ENSDARG00000102414	myhz1.1l	48.71	106.78	26.66	72.34	-0.6513	0.000271
ENSDARG00000055705	f5	8.17	21.58	4.41	14.4	-0.6614	0.008182
ENSDARG00000059558	tecta	3.23	11.01	0.66	8.31	-0.6668	0.00099
ENSDARG00000044319	fstl4	1.99	7.6	1.31	4.73	-0.667	0.038384
ENSDARG00000057568	nefla	1.42	4.84	0.47	3.47	-0.668	0.038118
ENSDARG00000037383	pax5	0	1.54	0	0.95	-0.6969	0.010793
ENSDARG00000040295	apoeb	181.75	450.46	102.61	285.35	-0.7045	8.94E-09
ENSDARG00000074376	mdga1	1.71	7.05	1.03	4.31	-0.7141	0.028939
ENSDARG00000104722	myl2a	13.77	10.46	7.6	6.94	-0.7368	0.041831
ENSDARG00000040306	otomp	8.07	32.03	0.28	23.76	-0.7382	0.029165
ENSDARG00000102456	cfhl4	24.02	56.47	11.73	36.38	-0.7425	0.000636
ENSDARG00000078522	si:ch211-80h18.1	21.27	61.21	0.28	48.99	-0.7433	0.049549
ENSDARG00000075803	slc41a2a	1.14	4.84	0.19	3.36	-0.7523	0.010261
ENSDARG00000037475	foxb2	0.47	3.3	0.19	2	-0.7836	0.026069
ENSDARG00000012694	c3a.1	74.64	195.07	41.4	114.71	-0.7888	2.75E-07
ENSDARG00000094041	krt17	362.65	227.87	201.08	140.57	-0.7895	1.50E-14
ENSDARG00000042221	mthfd1l	30.77	94.34	14.46	57.3	-0.8019	2.86E-08
ENSDARG00000043589	ca4a	1.23	5.61	0.56	3.36	-0.8031	0.023637
ENSDARG00000037281	fgg	39.03	76.62	14.93	49.31	-0.8482	0.001589
ENSDARG00000021004	c5	10.35	33.13	6.48	17.66	-0.8489	0.001838
ENSDARG00000070918	si:ch211-284e20.	37.22	86.42	13.14	54.15	-0.8777	2.84E-05
ENSDARG00000100442	cfh	22.32	64.18	11.36	35.12	-0.8961	3.62E-05
ENSDARG00000088589	ponzr3	5.6	2.42	2.72	1.58	-0.8993	0.021779
ENSDARG00000102050	mcoln3b	1.9	8.15	1.13	4.21	-0.9123	0.02746
ENSDARG00000016771	tfa	483.24	983.48	158.28	614.12	-0.9252	0.036889
ENSDARG00000014031	abcc2	49	75.74	17.37	48.26	-0.9265	0.046112
ENSDARG00000069583	cndp1	19.28	50.2	9.95	26.6	-0.9267	0.001517
ENSDARG00000033104	tmc2a	0.38	2.42	0	1.47	-0.9296	0.025726
ENSDARG00000005586	zbtb20	0.19	2.42	0	1.37	-0.9299	0.014593
ENSDARG00000055278	cfb	10.92	28.4	4.04	16.51	-0.9361	0.001614
ENSDARG00000015662	pla2g12b	47.76	110.96	19.81	62.77	-0.9426	3.49E-05
ENSDARG00000099425	cfi	14.15	39.08	6.95	20.61	-0.9497	0.001372
ENSDARG00000044685	nr0b2a	3.42	10.79	1.03	6.31	-0.9531	0.008288
ENSDARG00000093068	c3b.1	23.55	46.12	6.76	28.81	-0.9699	0.002405
ENSDARG00000008969	fgb	56.31	114.27	19.15	67.6	-0.9755	0.00024
ENSDARG00000041645	a2ml	94.2	239.21	38.3	130.27	-0.9839	1.85E-08
ENSDARG00000040298	apoa4b.1	138.16	381.22	58.02	202.39	-0.996	1.92E-11
ENSDARG00000018459	msrb2	15.95	41.83	8.45	20.29	-1.0075	0.007165
ENSDARG00000077872	LOC100006895	20.8	57.35	8.26	29.02	-1.0678	5.72E-05
ENSDARG00000012076	apoa1a	674.11	1956.06	280.31	969.8	-1.0731	2.18E-16
ENSDARG00000020741	fga	56.12	131.88	15.77	73.39	-1.0763	3.44E-06
ENSDARG00000089402	si:ch73-281i18.3	1.71	0.11	0.75	0.11	-1.0815	0.015041
ENSDARG00000053973	fetub	205.58	391.34	75.66	206.07	-1.0832	0.000208
ENSDARG00000022767	apobb.1	248.6	1618.44	135.75	744.49	-1.0848	6.58E-49
ENSDARG00000023111	plg	30.39	73.32	9.48	39.11	-1.0938	4.54E-05

ENSDARG00000105411	si:ch211-113d11.	12.25	31.48	4.32	16.09	-1.0993	0.002098
ENSDARG00000045516	itih2	26.02	66.05	8.64	34.28	-1.1011	3.50E-05
ENSDARG00000042780	apoba	59.35	316.93	24.31	150.66	-1.1047	8.57E-31
ENSDARG00000090286	serpina1	135.32	272.68	46.56	143.09	-1.1052	5.25E-05
ENSDARG00000036237	slc27a2a	37.41	101.06	15.87	48.15	-1.113	7.04E-06
ENSDARG00000042684	serpinc1	17.38	42.38	6.85	20.71	-1.1166	0.002759
ENSDARG00000059053	slc13a4	7.88	24.77	3.29	11.67	-1.126	0.002134
ENSDARG00000101324	apoa1b	986.9	2250.42	342.65	1134.87	-1.1316	3.19E-09
ENSDARG00000016319	c9	48.14	97.75	17.18	49.21	-1.1358	0.001411
ENSDARG00000103310	LOC108183319	1.61	0.11	0.66	0.11	-1.1595	0.021584
ENSDARG00000036041	f2	35.42	81.57	11.45	40.79	-1.1632	0.000125
ENSDARG00000053831	vtnb	21.56	53.39	8.07	25.34	-1.1656	0.000739
ENSDARG00000093774	rbp2b	21.08	53.28	8.17	24.6	-1.1822	0.000832
ENSDARG00000063518	zgc:153913	8.17	20.7	2.07	10.51	-1.1984	0.004422
ENSDARG00000092170	apoc1	96.67	338.95	42.15	146.98	-1.2037	2.48E-15
ENSDARG00000102241	zgc:136410	6.55	19.59	1.22	9.99	-1.2215	0.000601
ENSDARG00000092155	apoc2	95.91	234.81	37.18	104.3	-1.225	8.00E-07
ENSDARG00000041569	ces2a	86.13	188.46	26.29	89.47	-1.2461	9.28E-06
ENSDARG00000099200	zgc:123103	92.96	219.4	32.11	96.94	-1.2753	1.46E-06
ENSDARG00000104540	cyp2aa8	12.15	32.47	5.26	13.14	-1.278	0.013723
ENSDARG00000102004	apoea	113.38	298.99	38.68	130.79	-1.2829	1.85E-09
ENSDARG00000055388	vtna	12.34	36.99	4.69	15.56	-1.2845	0.000733
ENSDARG00000089874	pcdh2aa3; pcdh2	0.66	5.83	0.56	2.1	-1.2868	0.026874
ENSDARG00000015866	apoa2	829.56	1659.61	248.87	757.95	-1.3059	0.012449
ENSDARG00000004296	ambp	38.93	86.64	15.4	35.12	-1.3136	0.004765
ENSDARG00000024160	hao1	22.13	46.57	7.23	20.19	-1.3251	0.012432
ENSDARG00000103277	cyp24a1	13.86	4.18	5.07	2.1	-1.3312	2.81E-05
ENSDARG00000104207	pcyt1bb	0.85	7.49	0.47	2.84	-1.3332	0.0042
ENSDARG00000010312	cp	43.87	98.41	11.45	42.79	-1.3913	6.73E-05
ENSDARG00000095863	afp4	71.12	233.27	19.62	89.68	-1.4776	3.58E-13
ENSDARG00000076838	apom	24.59	73.87	6.38	24.5	-1.6729	4.90E-06
ENSDARG00000094929	apoa4b.3	0.38	4.84	0.09	1.37	-1.8381	0.00736
ENSDARG00000034403	zgc:173443	0.57	5.17	0	0.74	-2.9555	0.013148
ENSDARG00000092419	vtg7	0.57	10.02	0	1.16	-3.1905	0.000239

Supplementary Table S3. The real-time PCR and construction primers.

The real-time PCR primers

Gene	Sequence(5'to3')
zebrafish-*cyp7a1* -FP	GCAAGATTGCTGGGCAGTATG
zebrafish-*cyp7a1* -RP	TCATGACCAAATGCCTTCGCA
zebrafish-*apoa1a* -FP	TCTCTTGGCCTTGGGTTCC
zebrafish-*apoa1a* -RP	GCTCATAGTCGGTTCCGTCC
zebrafish-*apoa1b* -FP	CACCGTATTCCTGGCAGGTT
zebrafish-*apoa1b* -RP	CTGGCCCAGGAAGACTTTGTA
zebrafish-*apoa2* -FP	CTTGCACTCCAAGTGTCAGT
zebrafish-*apoa2* -RP	CCCAAGCATTGACAAGCCTC
zebrafish-*apoea* -FP	AAGAGGCCGTGGATCAGTTC
zebrafish-*apoea* -RP	GCTGTCAGAGATCAGTGTGTCTA
zebrafish-*apoeb* -FP	TGACATGACCGACGCTAAGG
zebrafish-*apoeb* -RP	TGTAGGTTGCTACGGTGTTGC
zebrafish-*fga* -FP	ACCGATGATGACTGGGGAAG
zebrafish-*fga* -RP	CCACCAGGAGACGTCGAATC
zebrafish-*fgb* -FP	CTCCAGGCCCTGTTTCTCAG
zebrafish-*fgb* -RP	GGGCACAACACACCCATTTT
zebrafish-*fgg* -FP	GTACTTCAGAGGAGGCGGGA
zebrafish-*fgg* -RP	TCCTCAGCACATAAGGCACG
zebrafish-*cfb* -FP	CCTGGTTGGCACAGCTTAGT
zebrafish-*cfb* -RP	ACCTTTTCCACTTTTACATCCGTG
zebrafish-*cfh* -FP	CGGTTCCAAACTGCATAGAGAT
zebrafish-*cfh* -RP	GAGGCACATCTGGGAAATGC
zebrafish-*cfi* -FP	CTTTGCGATGGTATTGACGA
zebrafish-*cfi* -RP	CTCCAGCTCGTCTTCTCCAC
zebrafish-*serpinc1* -FP	GGAAATACAACACCGGGCAAG
zebrafish-*serpinc1* -RP	TGGGCTTTGCGTTGCAGATA
zebrafish-*hnf4a* -FP	CCTGGCAAATGATCGAGCAA
zebrafish-*hnf4a* -RP	GGTGGAGAGAATGATGAGCG
zebrafish-*tp53* -FP	GGAGAGGAGGTCGGCAAAAT
zebrafish-*tp5* 3-RP	CCTGGGGGCTGAATACTTATCAA
zebrafish-*wee1* -FP	CCCACACCCCAAAGAGCTTA
zebrafish-*wee* 1-RP	ACCGTCCAGACAGGCTTTAC
zebrafish-*gapdh* -FP	GAGGCTTCTCACAAACGAGGA
zebrafish-*gapdh* -RP	TGGCCACGATCTCCACTTTC

The construction primers.

Gene	Sequence(5'to3')
human-*YPEL5* -FP	CCGGAATTCGGCAGAATTTTCCTTGATCAT
human-*YPEL5* -RP	CCGCTCGAGTCAAGAGTTATCAGATGGTAC
zebrafish-*hnf4a* -FP	CCGGAATTCGAGATGGCAGACTATAGCGAG
zebrafish-*hnf4a* -RP	CCGCTCGAGTCAGATGGCCTCTTGTTTAGT
mouse-*Ppara* -FP	CGGGATCCATGGACTACAAGGACGACGATGACAAAATGGTGGACACAGAGAGC
mouse-*Ppara* -RP	CCGCTCGAGTCAGTACATGTCTCTGTAGATCTCTTGCAA
mouse-*Hnf4a* enhancer-FP	CCGAGCTCGTCCACGCCATTCATGTCTC
mouse-*Hnf4a* enhancer-RP	CCGCTCGAGCCTGAAGCTGGAACCCGATA
